# Prognosis and Treatment of Plasmablastic Lymphoma in the United States: A Multicenter Retrospective Study

**DOI:** 10.21203/rs.3.rs-7607922/v1

**Published:** 2025-09-30

**Authors:** Matthew Hamby, Brian Egleston, Zachary Frosch, Ralphael Steiner, Ariela Noy, Veronica Carvajal, Emeline Chong, Seda Tolu, Gaston Jean-Louis, Jennifer Amengual, Romil Patel, Sairah Ahmed, John Sharp, Timothy Voorhees, Robert Baiocchi, Andres Ramirez-Gamero, Jorge Castillo, Emily Hamburger, Christopher Dittus, Imran Nizamuddin, Neha Mehta-Shah, Nyein Nyein Thaw Dar, Jose Sandoval-Sus, Alexandra Rojek, Peter Riedell, Niloufer Khan, Alexey Danilov, Vinod Solipuram, Paul Rubinstein, Anthony Ariotti, Gita Suneja, Alexander Vartanov, Charles Milrod, Adam Olszewski, Gordon Smilnak, Emily Ayers, Sahaj Desai, Joanna Rhodes, Gabriella Magarelli, Tatyana Feldman, Meredith Pellon, David Aboulafia, William Bae, Carlos Galvez, Vineel Bhatlapenumarthi, Mehdi Hamadani, Stefan Barta

**Affiliations:** New York Presbyterian / Weill Cornell Medical Center; Fox Chase Cancer Center; Fox Chase Cancer Center; Memorial Sloan Kettering Cancer Center; Memorial Sloan-Kettering Cancer Center; Abramson Cancer Center, University of Pennsylvania; University of Pennsylvania; Columbia University Irving Medical Center; Columbia University Irving Medical Center; Columbia University Irving Medical Center; The University of Texas MD Anderson Cancer Center; The University of Texas M.D. Anderson Cancer Center; The Ohio State University; The Ohio State University; The Ohio State University; Dana-Farber Cancer Institute; Dana-Farber Cancer Institute; Lineberger Comprehensive Cancer Center, University of North Carolina; Lineberger Comprehensive Cancer Center, University of North Carolina; Siteman Cancer Center, Washington University in St. Louis; Siteman Cancer Center, Washington University in St. Louis; Moffitt Cancer Center at Memorial Healthcare System; Moffitt Cancer Center at Memorial Healthcare System; University of Chicago; University of Chicago; City of Hope National Medical Center; City of Hope; John H. Stroger Jr. Hospital of Cook County; John H. Stroger Jr. Hospital of Cook County; Huntsman Cancer Institute, University of Utah; Huntsman Cancer Institute, University of Utah; Fox Chase Cancer Center; Brown University; The Warren Alpert Medical School of Brown University; University of Virginia; University of Virginia; Rutgers Cancer Institute of New Jersey; Rutgers; John Theurer Cancer Center, Hackensack University Medical Center; John Theurer Cancer Center, Hackensack Meridian Healt; Virginia Mason Medical Center; Virginia Mason Medical Center; University of Illinois at Chicago; University of Illinois at Chicago; Medical College of Wisconsin; Medical College of Wisconsin; University of Pennsylvania

## Abstract

Plasmablastic lymphoma (PBL) is a rare, aggressive AIDS-related lymphoma observed in patients with immunosuppressed states as well as in immunocompetent individuals. We sought to determine survival outcomes, prognostic factors, and optimal treatment regimens in a large, contemporary cohort of patients with PBL in the United States. We performed a multicenter, retrospective cohort study, including 344 patients diagnosed with PBL between 2005 and 2022. Patients were stratified into cohorts according to underlying immune status. Survival outcomes were calculated using Kaplan-Meier statistics, with cohort-specific survival outcomes adjusted using propensity score-based weighting. Factors associated with outcomes were assessed via multivariable models using multiple imputation. The median age at diagnosis was 53 years, most patients were male (n = 270), and many had HIV (n = 164). The median OS was 5.0 years, with a median PFS of 1.4 years. Patients living with HIV had the best outcomes, whereas patients with prior organ transplantation had the worst outcomes. Use of higher intensity chemotherapy regimens and use of a proteasome inhibitor in the frontline setting did not show survival benefit. While there was no clear optimal treatment approach in the frontline setting, the median OS of 5.0 years is dramatically improved compared with historical controls.

## Introduction

Plasmablastic lymphoma (PBL) is a rare subtype of large B-cell lymphoma first described in 1997 in a small cohort of people living with HIV (PLWH).^1^ In this initial cohort, it presented as a tumor in the oral cavity and was almost uniformly fatal. Since then, this entity has been identified post-transplant, in the setting of other immunosuppressed states, and even in otherwise immunocompetent patients.^2^

PBL arises from the post-germinal center plasmablast, an activated B cell that ranges from immunoblastic to plasmacytic in morphology.^3^ Immunophenotypically, PBL is characterized by expression of plasma cell markers (CD38, CD138) and most commonly a lack of B-cell markers (CD19, CD20, PAX5).^4^ *MYC* rearrangement and EBV infection are believed to contribute to the pathogenesis of PBL, but their roles remain incompletely elucidated.^3^

While first described as a lesion in the oral cavity/jaw,^1^ PBL also presents in nodal and other extranodal sites. Many patients are diagnosed with advanced-stage disease that follows an aggressive clinical course.^5^ While the prognosis of PBL was initially reported to be dismal, with a median overall survival of 8–15 months, recent studies have suggested improved outcomes.^6,7^ The mainstay of initial management has been multiagent cytotoxic chemotherapy. Using CHOP (cyclophosphamide, doxorubicin, vincristine, and prednisone) and CHOP-like regimens, 1- and 2-year overall survival of 50–60% has been reported in small case series (n = 35).^8^ The addition of biological agents commonly used for the management of plasma cell disorders, such as the proteasome inhibitor bortezomib or the CD38-directed monoclonal antibody daratumumab, may confer some benefit, but has not yet been tested in a randomized fashion.^9,10^ Moreover, no well-defined standard of care exists. While NCCN guidelines consider CHOP to be inadequate and favor higher intensity regimens,^11^ recent studies have called this into question.^6,12,13^

Given its rarity, the literature on PBL is primarily limited to case reports and small case series, with larger multicenter and database studies appearing in recent years.^5,12^ Still, significant unknowns remain surrounding the underlying biology and treatment of PBL. We aimed to perform a large, multicenter, retrospective study to understand disease characteristics, prognostic factors, and treatment-related outcomes in a contemporary cohort of PBL patients treated in the United States.

## Methods

### Study Design

**Figure 1:**
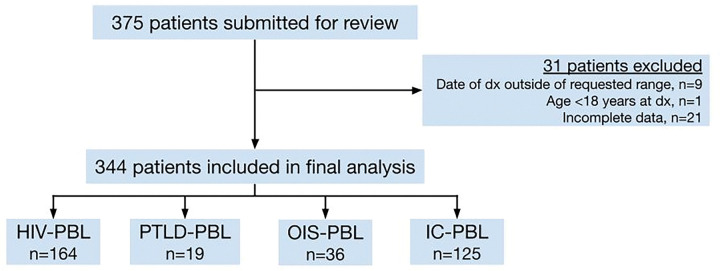
Consort Diagram HIV-PBL: PBL in a person living with HIV; PTLD-PBL: PBL arising as a post-transplant lymphoproliferative disorder; OIS-PBL: PBL arising in the setting of some other immunosuppressed state; IC-PBL: PBL in an otherwise immunocompetent patient.

In this multicenter, retrospective cohort study, we identified 344 patients with PBL from 21 academic centers in the United States. Each center identified patients retrospectively using electronic medical records and submitted their anonymized data to the study center. Patients were included if they were ≥ 18 years old and diagnosed with PBL between 1/2005 and 12/2022. Patients were excluded if their diagnosis failed to meet the World Health Organization (WHO) diagnostic criteria^14^ for PBL or their data were deemed incomplete. Patients included in the final analysis were grouped into one of four cohorts according to immune status (Fig. 1): patients with a previous history of HIV or HIV diagnosed concurrently with PBL were classified as HIV-PBL; patients with prior organ transplantation were classified as PBL arising as a post-transplant lymphoproliferative disorder (PTLD-PBL); those without HIV or prior transplant, but with known immunosuppression (e.g. underlying lymphoproliferative disorder, previous chemotherapy for malignancy, primary immunodeficiency, iatrogenic immunodeficiency, or autoimmune disease on current or prior immunosuppressive therapy with prednisone +/− biologic agents) were classified as PBL arising in the setting of other immunosuppressed states (OIS-PBL). All remaining patients who did not meet the criteria mentioned above were classified as immunocompetent PBL (IC-PBL).

### Objectives and Definitions

The primary objective of this study was to determine the overall survival (OS) of the entire cohort. OS was defined as the time from PBL diagnosis to death from any cause or censoring at the time of last follow-up (FU). Secondary objectives included determining progression-free survival (PFS), non-relapse mortality (NRM), and treatment-related mortality (TRM), as well as assessing the prognostic impact of patient-, disease-, and treatment-related factors on survival outcomes. PFS was defined as the time from initiation of treatment to relapse/progression, death from any cause, or censoring at time of last FU. TRM was defined as death not due to PBL within 30 days of the most recent treatment.

Patients were staged with CT or combined PET/CT. Bone marrow involvement was assessed either via bone marrow biopsy or PET/CT as per institutional practice. Involvement of the oral cavity/jaw, nasopharynx, oropharynx, paranasal sinuses, or orbit was defined as disease occurring in the head and neck. Immunophenotype was characterized by immunohistochemistry (IHC). EBV status was assessed either via IHC for latency membrane protein-1 (LMP1) or in situ hybridization (ISH) to EBV-encoded RNA (EBER). *MYC* rearrangement was assessed via fluorescence in situ hybridization (FISH), with rearrangement at any locus considered a positive result.

As detailed in **Supplemental Table 1**, we categorized chemotherapeutic backbone regimens received in the frontline (1L) setting into four groups: 1) standard-intensity CHOP/CHOP-like regimens, 2) dose-adjusted etoposide, prednisone, vincristine, cyclophosphamide, and doxorubicin (EPOCH), 3) high intensity regimen with hyperfractionated cyclophosphamide, vincristine, doxorubicin, dexamethasone, methotrexate, and cytarabine (Hyper-CVAD) or cyclophosphamide, vincristine, doxorubicin, high-dose methotrexate/ifosfamide, etoposide, and high-dose cytarabine (CODOX-M/IVAC), and everything else as 4) “other”. Second-line (2L) chemotherapeutic regimens are also detailed in **Supplemental Table 1**.

### Statistical Analysis

Continuous variables were summarized using medians with ranges and compared using the Wilcoxon rank-sum test. Categorical variables were summarized using frequencies and compared using the **χ**^2^ test.

Unadjusted OS and PFS were calculated via the Kaplan-Meier method. Cox regressions were used to investigate variables associated with survival. Disease classification-specific survival figures were adjusted by inverse generalized propensity score-based weighting.^15,16^ The PTLD-PBL group was excluded from the propensity score models due to the small sample size. Factors included in multivariable Cox regression and the multinomial logistic propensity score model included age, sex, year of diagnosis, race, ethnicity, immune status, ECOG performance status, Ann Arbor stage, extranodal disease, LDH elevation, IPI score, *MYC* rearrangement, EBV positivity by either LMP1 or EBER, 1L chemotherapeutic regimen, use of a proteasome inhibitor (PI) in the 1L, and use of CNS prophylaxis (PPx). The impact of consolidative autologous stem cell transplant (ASCT) was assessed in a separate sensitivity analysis. We used multiple imputation to account for missing data in the multivariable models.^17^

Approval for this study was granted by the Institutional Review Board (IRB) of The University of Pennsylvania (Philadelphia, USA) and by the IRBs of all participating institutions.

## Results

### Patient characteristics

Three hundred seventy-five patients were identified from 21 institutions. After excluding 31 patients who did not meet eligibility criteria, 344 patients were included in the final analysis: 48% had HIV-PBL (n = 164), 6% had PTLD-PBL (n = 19), 10% had OIS-PBL (n = 36), and 36% had IC-PBL (n = 125) (Fig. 1). The median age at diagnosis was 53 years (range 19–91) (Table 1). Most patients were male (78%, n = 270); 67% of patients were White (n = 230), 17% were Black/African American (n = 59), and 33% were Hispanic/Latino (n = 114). The median age at diagnosis was significantly younger in the HIV-PBL cohort (46 years) compared with the PTLD-, OIS-, and IC-PBL cohorts (55, 67, and 68 years, respectively; p < 0.001).

In the HIV-PBL cohort, the median CD4 count at time of PBL diagnosis was 147 cells/μL (range 1–986); 30% (n = 49) had a CD4 count < 100 cells/μL. Fifty-eight percent (n = 95) of patients were previously on antiretroviral therapy (ART) with a median CD4 count of 177 cells/μL. Thirty-six percent (n = 59) were ART-naïve and commenced ART at the time of PBL diagnosis, with a median CD4 count of 102 cells/μL.

In the PTLD-PBL cohort, the median time from transplant to PBL diagnosis was 7.9 years (range 1–25). Of the 36 patients classified as having OIS-PBL, roughly half (n = 17) had an underlying lymphoproliferative disorder (LPD) (CLL, n = 6; FL, n = 3; DLBCL, n = 3; MZL, n = 2; WM, n = 1; HL, n = 1; MALT Iymphoma, n = 1), with a median time from diagnosis of LPD to PBL of 12 years (range 6–26).

Most patients were diagnosed with advanced-stage PBL [stage III (n = 22, 6%) or IV (n = 227, 66%)]. The majority had nodal disease (n = 215, 63%), and most had extranodal involvement (n = 316, 92%), with the most common extranodal site being the GI tract (n = 107, 31%). Bone marrow involvement was identified in 30% of patients who underwent a bone marrow biopsy (n = 63/212). Only 3% of patients presented with CNS involvement (n = 11). Twenty-seven percent presented with disease located in the head and neck (n = 94), with significantly more in the HIV-PBL cohort compared with the PTLD-, OIS-, and IC-PBL cohorts (32%, vs 11%, 8%, and 29%, respectively; p = 0.008).

The most common immunophenotype was CD38+ (84%), CD138+ (87%), CD19− (83%), and CD20− (90%). Fifty-eight percent of tested samples had a detectable *MYC* rearrangement (n = 45/78), and this genomic abnormality was significantly more common in the HIV-PBL cohort at 74% (n = 26/35; p = 0.045). Likewise, 64% of tested samples were EBV + by EBER ISH, with the OIS- and IC-PBL cohorts significantly less likely to be EBER + compared with the HIV- and PTLD-PBL cohorts (p < 0.001).

### Survival Outcomes

Median time until death or censoring for the entire cohort was 3.4y (range 0–17). Median OS was 5.0y (95% CI: 3.2–8.6), with 1y- and 2y- OS rates of 70% and 59%, respectively. Median PFS was 1.4y (95% CI: 1.0–3.0), with 1y- and 2y- PFS rates of 54% and 47%, respectively. The median OS after 1st relapse/progression was 0.6y (95% CI: 0.42–0.85). There were 170 deaths. The most common cause of death was PBL (n = 104), followed by infection (n = 25, with 12 being classified as TRM), and second primary malignancies (NSCLC, n = 2; HNSCC, n = 1; esophageal carcinoma, n = 1; mucinous adenocarcinoma, n = 1). The cause of death was unknown in 29 patients.

**Figure 2:**
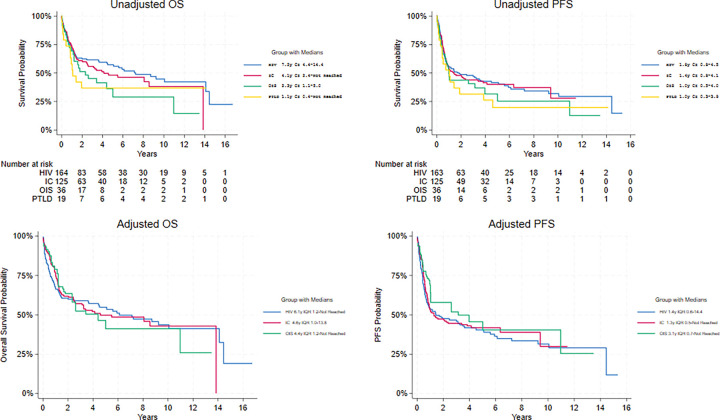
Survival Outcomes by Immune Status Cohort Kaplan-Meier curves comparing: unadjusted overall survival by immune status (2a); unadjusted progression-free survival by immune status (2b); propensity score-adjusted overall survival by immune status (2c); propensity score-adjusted progression-free survival by immune status (2d). Median survival (years) with 95% CI is denoted next to each curve, with p-values representing significant difference in median survival between cohorts.

When comparing survival outcomes by immune status (Fig. 2a–b), the PTLD-PBL cohort had the poorest outcomes, with a median OS of 1.1y (95% CI: 0.43-not reached) and a median PFS of 1.0y (95% CI: 0.3–3.9). In contrast, the HIV-PBL cohort had the best outcomes, with a median OS of 7.2y (95% CI: 4.4–14.4) and a median PFS of 1.8y (95% CI: 0.8–4.5). Median OS was 2.3y (95% CI: 1.1–5.0) with a median PFS of 1.0y (95% CI: 0.5–4.0) in the OIS-PBL cohort. For the IC-PBL cohort, median OS was 4.1y (95% CI: 2.4-not reached) with a median PFS of 1.4y (95% CI: 0.8–4.1).

### Propensity Score Adjusted Survival

We present unadjusted and propensity score-adjusted characteristics of the HIV-PBL, IC-PBL, and OIS-PBL cohorts in **Supplemental Table 2**.

Propensity score-adjusted OS medians for the non-PTLD-PBL cohorts were: 6.1 years (Interquartile Range [IQR]: 0.6-not reached) for HIV-PBL, 4.6 years (IQR: 1.0–13.8) for IC-PBL, and 4.4 years (IQR: 1.2-not reached) for OIS-PBL (Fig. 2c). Propensity score-adjusted Cox regressions did not find significant OS differences among the three groups (HR = 1.0, 95% CI: 0.6–1.6 for IC-PBL; HR = 1.1, 95% CI: 0.6–2.0 for OIS-PBL, relative to the HIV-PBL cohort). Adjusted PFS medians were 1.4 years (IQR: 0.4–14.4) for HIV-PBL, 1.3 years (IQR: 0.5-not reached) for IC-PBL, and 3.1 years (IQR: 0.7-not reached) for OIS-PBL (Fig. 2d). Propensity score-adjusted PFS models did not find significant differences among groups (HR = 0.9, 95% CI: 0.6–1.4 for IC-PBL; HR = 0.8, 95% CI: 0.4–1.5 for OIS-PBL, relative to HIV-PBL).

**Figure 3:**
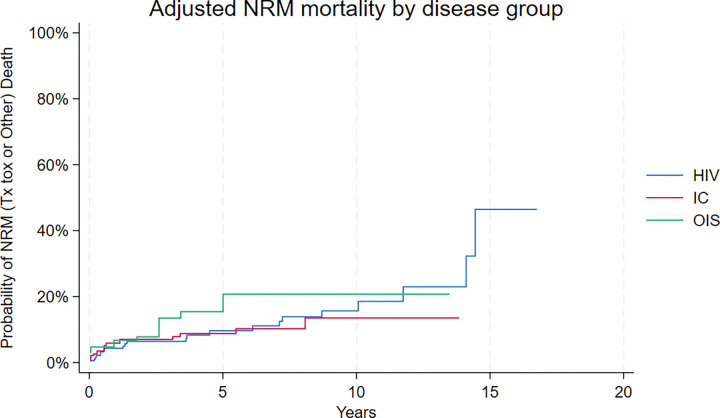
Propensity Score Adjusted Non-Relapse Mortality_(NRM)_by Immune Status Cohort Cumulative incidence of death due to causes other than PBL, by immune status cohort.

Adjusted median NRM was not reached for any of the three cohorts (Fig. 3). Twenty-fifth percentile mortality was 14.4 years for HIV-PBL, and not reached for the IC-PBL or OIS-PBL cohorts. Subdistribution hazard ratios were 1.0 (95% CI: 0.6–1.5) for IC-PBL and 0.9 (95% CI: 0.5–1.6) for OIS-PBL, relative to HIV-PBL.

### Sensitivity Analysis

Substantial differences between the cohorts impeded our ability to achieve an optimal balance of demographic and clinical factors among the three adjusted arms (**Supplemental Table 2**). For example, the oldest patient was 72 in the HIV-PBL cohort versus 91 in the IC-PBL cohort. To ensure that residual confounding was not driving our findings, we estimated a logistic propensity score model using data only from patients in the HIV-PBL and IC-PBL cohorts who were 73 or younger. The OIS-PBL cohort was excluded from this analysis due to its small sample size. In this sensitivity analysis, we achieved better adjusted demographic balance across groups, and the results (**Supplemental Table 3**) were similar to those of the three-group propensity score-adjusted model.

### Prognostic Factors

The results of the survival analysis are detailed in Table 2. In the multivariable Cox regression model utilizing multiple imputation methods, only advanced age, ECOG ≥2, advanced stage, and LDH elevation were associated with worse OS. We sought to more thoroughly assess the impact of several factors on survival outcomes, including CD4 count and *MYC*/EBV status. In the HIV-PBL cohort, a CD4 count ≥ 100 cells/μL at the time of PBL diagnosis was not associated with improved OS on multivariable analysis. Likewise, initiation of ART at the time of PBL diagnosis impacted neither OS nor PFS within the HIV-PBL cohort. Further, we assessed the impact of *MYC* translocation and EBV status on survival outcomes in the entire cohort: neither was an independent predictor of OS; however, EBV positivity was associated with improved PFS (HR = 0.57, 95% CI: 0.39–0.84, p = 0.004). The data on *MYC* rearrangement should be precautionary, as only 23% of patients were assessed for this abnormality.

### Frontline Treatment

Of the 313 patients who received chemotherapy in the frontline setting, most received EPOCH-based regimens (70%; n = 220), 14% received CHOP/CHOP-like regimens (n = 44), and only 8% received Hyper-CVAD or CODOX-M/IVAC (n = 13, each). Many patients received agents in addition to chemotherapy in the 1L setting, with 34% receiving bortezomib (n = 105), 19% receiving rituximab (n = 60), and 4% receiving daratumumab (n = 12). Three patients received single-agent biologic treatment (bortezomib, n = 2; rituximab, n = 1). There was no apparent benefit to either OS or PFS with the use of EPOCH or other high-intensity chemotherapy regimens over standard intensity CHOP/CHOP-like regimens in both the univariable and multivariable models (Table 2). This lack of OS and PFS benefit with use of EPOCH (compared with CHOP) was observed in a separate subgroup analysis of the HIV-PBL cohort as well. The overall and complete response rates (ORR, CRR) associated with EPOCH were 64% and 52%, respectively, versus 66% and 52% for CHOP. Of note, EPOCH was associated with higher rates of TRM than CHOP: 4% of EPOCH-treated patients died of TRM (n = 8) vs 2% of CHOP-treated patients (n = 1), p < 0.001. Furthermore, the use of a proteasome inhibitor failed to show either OS or PFS benefit on MVA (OS: HR = 0.99, 95% CI: 0.68–1.43, p = 0.950; PFS: HR = 1.10, 95% CI: 0.79–1.53, p = 0.591).

### Radiation, Autologous Stem Cell Transplant, and CNS Prophylaxis

Eighteen percent of patients (n = 63) received RT as consolidation in the 1L setting, while 8% were consolidated with a hematopoietic ASCT at first remission (n = 27). Both RT and ASCT consolidation were independently associated with improved OS on UVA (Table 2); however, only ASCT remained associated with improved OS (HR = 0.43, 95% CI: 0.19–0.97, p = 0.041) and PFS (HR = 0.49, 95% CI: 0.26–0.94, p = 0.032) when added to the multivariable Cox model in a separate sensitivity analysis.

Thirty-three percent of patients (n = 113) received CNS prophylaxis in the form of intrathecal methotrexate/cytarabine (MTX/AraC; n = 83), high-dose MTX (n = 23), or both (n = 7). None of these treatment modalities were associated with improvements in OS or PFS in the multivariable Cox regression models. Only one of the patients who received CNS PPx (n = 113) experienced relapse involving the CNS, versus three of the patients who did not receive CNS PPx (n = 231).

### Second-Line Treatment

Of the 138 patients who relapsed or progressed after receiving frontline treatment, 105 were treated with 2L therapies. The most commonly used 2L regimens included ICE (ifosfamide, carboplatin, and etoposide, n = 30), DHAP/similar (dexamethasone, high-dose cytarabine, and platinol, n = 23), low-intensity myeloma-like regimens (n = 19), and GemOx/similar (gemcitabine and oxaliplatin, n = 8) (**Supplemental Table 1**). The ORRs for each of these regimens were: 50%, 39%, 26%, and 38%, respectively.

Furthermore, 51% of these 105 patients were treated with biological agents alone or in addition to chemotherapy, such as daratumumab (n = 24), bortezomib (n = 27), rituximab (n = 9), or the immunomodulatory drug lenalidomide (n = 13). Several patients were consolidated in the second-line setting with either an autologous (n = 3) or allogeneic (n = 3) hematopoietic cell transplant (HCT). Of note, four patients received chimeric antigen receptor (CAR) T-cell therapy in the 2L setting (CD19-directed, n = 3; unknown target, n = 1), with only one alive at last FU (treated with lisocabtagene maraleucel).

## Discussion

We performed a multicenter, retrospective study to better understand the underlying biology, survival outcomes, and optimal treatment of PBL in a contemporary cohort of patients in the US. To our knowledge, this is the largest real-world cohort of PBL patients to date.

The main finding of this study was the markedly improved median OS in our cohort (5.0y) compared with a recently published international cohort (1.4y), albeit with similar median PFS (1.4y vs 8.4 mo).^12^ This discrepancy may be explained by differences in second-line therapies available during the time frame of the two studies. Di Ciaccio et al. enrolled from 1999 to 2020, with only 35% of patients diagnosed after 2015, whereas we enrolled from 2005 to 2022, with 70% diagnosed after 2014. It is possible that increased use of modern biological agents in our more contemporaneous cohort explains the improvement in OS.

The results of our study are broadly consistent with the current understanding of PBL. Known lymphoma risk factors that are usually captured in the IPI, such as advanced age, stage III/IV, ECOG ≥ 2, and LDH elevation, were associated with poor OS in our cohort.^18^ Consistent with Di Ciaccio et al., we also found EBV negativity to be associated with poor PFS.^12^

While there is mixed evidence regarding the prognostic impact of *MYC* rearrangement in PBL,^12,13,19,20^ our results suggest that *MYC*+/EBV− PBL may represent an aggressive disease subtype; however, this requires further exploration, especially as data on *MYC* rearrangement was missing in many patients. EBV-positivity has been associated with different genetic features in Burkitt lymphoma (BL) when compared to EBV-negative cases. Recently, investigators identified that EBV + BL is characterized by fewer driver mutations, higher aberrant somatic hypermutation rates, and significantly more breakpoints upstream of *MYC*.^21^ Therefore, interaction of EBV and *MYC* in PBL may result in different pathobiology underlying the observed differential outcomes. Furthermore, the association between female sex and poor OS on UVA (albeit with no association on MVA) is intriguing and warrants further investigation, particularly since males tend to have worse survival outcomes in most other lymphoma subtypes.^22,23^ However, this observation may be related to the fact that male sex was predominant in the HIV-PBL cohort, which, in general, had better outcomes.

Despite improvement in median OS, PBL remains a difficult-to-treat disease, as demonstrated by a 5-year OS rate of 50% in our cohort. Survival outcomes differed by immune status: HIV-PBL had the best outcomes (median OS 7.2y, median PFS 1.8y), while PTLD-PBL had the worst outcomes (median OS 1.1y, median PFS 1.0y). This may be due to a partially reversible immunodeficiency in PLWH, especially as about a third of the HIV-PBL cohort was previously ART-naïve. The poor survival outcomes of PTLD-PBL suggest this may represent an aggressive subtype, possibly related to the mostly irreversible, underlying, severe iatrogenic immunodeficiency in post-transplant patients. The underlying histopathologic features of this entity represent an area of future investigation. While there were various underlying immunosuppressed states in the OIS-PBL cohort, many were LPDs, raising the possibility that the PBL diagnosis represented a transformation event. Further investigation into the biology and clinical outcomes of this subgroup is warranted.

The optimal treatment of PBL remains poorly defined due to the rarity of the disease and paucity of prospective clinical trials. While early studies of HIV-associated non-Hodgkin lymphomas suggested superior outcomes with EPOCH compared with CHOP and prompted NCCN guidelines to favor higher-intensity regimens, more recent retrospective studies have increasingly suggested no benefit to such an approach in PBL.^11,12,24,25^ Our results similarly found no benefit with the use of EPOCH or other high-intensity regimens in the 1L setting over CHOP and therefore do not support the use of higher-intensity regimens, which are subject to higher rates of treatment-related morbidity and mortality. However, none of these are randomized prospective studies.

Similarly, despite promising results in small, retrospective case series,^7^ proteasome inhibitors failed to show frontline benefit in a recent multicenter study,^12^ consistent with the findings in our cohort. Nevertheless, our study was not powered to definitively address the merit of biological agents classically used for plasma cell malignancies, such as bortezomib, daratumumab, and lenalidomide, in the management of PBL. However, the improved OS despite a similar PFS after 1L therapy in our US cohort could indicate a role for these agents in PBL. Prospective and ideally randomized clinical trials will be necessary to address this question. At present, a prospective randomized clinical trial in the US explores the combination of daratumumab with EPOCH,^26^ while another study sponsored by the Fondazione Italiana Linfomi studies the combination of daratumumab, bortezomib, and dexamethasone for relapsed/refractory (r/r) PBL.^27^

While there is no superior chemotherapeutic regimen in the 1L setting, RT and ASCT consolidation in our study were associated with improvements in OS, albeit with the inherent bias that to receive these treatments most patients would have needed first to achieve a complete response to initial therapy. However, as only 8% of the entire cohort underwent consolidative ASCT in first remission, the benefit of ASCT should be interpreted with caution and considered on a case-by-case basis in select, high-risk patients.

In contrast, the use of CNS prophylaxis was not associated with OS and had no apparent effect on the risk of CNS recurrence, although the numbers were too small for firm conclusions. This is in line with recent observations in DLBCL that indicate a lack of benefit of CNS prophylaxis irrespective of modality.^28,29^ Thus, the decision to use CNS prophylaxis in PBL should be highly individualized.

Several small studies have investigated the management of r/r PBL with chemotherapeutic regimens such as ICE, with or without agents like daratumumab and lenalidomide.^30,31,32,33^ We gathered data on 105 patients who underwent treatment in the r/r setting, and a comparison of survival outcomes using various 2L regimens, as well as the impact of use of biologic agents in the 2L setting will be addressed in future analyses. Further, while CAR T-cell therapy has revolutionized the treatment landscape of both DLBCL and multiple myeloma, its use in PBL has been limited. To our knowledge, there are only three published cases using CAR T-cell therapy in r/r PBL. One achieved a complete metabolic response, followed by disease progression at 5 months post-CD19-directed CAR T-cell infusion.^34^ Two additional patients achieved complete remissions in response to BCMA-directed CAR T-cell products, with CR documented at 6 and 14 months post-infusion, respectively.^35,36^ Herein, we report four patients treated with CAR T-cell therapy (CD19-directed, n = 3; unknown target, n = 1) with disappointing outcomes. It is unclear if the expression of CD19 and/or BCMA played a role in treatment choice. More data is needed regarding the safety, efficacy, and optimal target antigen of CAR T-cell therapies in the management of r/r PBL.

There are several limitations to this study. Namely, given the retrospective nature of data collection, there was considerable variation in data availability between participating institutions. While some clinicopathologic variables were missing, we addressed missing data bias by using multiple imputation for multivariable analyses. Further, no centralized pathologic review was done to confirm a diagnosis of PBL in each patient; however, all participating institutions were major academic centers with experienced hematopathology departments.

Despite the limitations of our study, it has several strengths, including our large sample size and granular, patient-level data. This allowed us to control for possible confounders by using propensity score adjustment and therefore significantly reduce bias. Further, this study expands the existing literature on this rare entity, augments findings of a recently published cohort from Australia, the UK, Canada, and Singapore, and at the same time contrasts them with our contemporary US cohort.^12^

The prognosis of PBL has significantly improved over recent decades, particularly for HIV-PBL. While no optimal treatment has been established, more intense cytotoxic regimens may not confer a significant survival benefit. Although biological agents commonly used for plasma cell dyscrasias have been suggested to be of benefit based on small case series, their frontline use was not associated with improved outcomes in our study, and their role in PBL remains to be defined. This can only be done definitively in well-designed, prospective, and randomized clinical trials. Better understanding of the pathogenesis that underlies PBL based on oncogenic events and interactions with the host immune environment may also lead to more rationally targeted therapies and further improved outcomes.

## Supplementary Material

Supplementary Files

This is a list of supplementary files associated with this preprint. Click to download.

• SupplementalTablesandFigures.pdf

## Figures and Tables

**Table 1.** Patient Characteristics, Clinical Presentation, and Treatment Patterns by Immune Status Cohort

	Total	HIV-PBL	PTLD-PBL	OIS-PBL	IC-PBL	
	n = 344 (%)	n = 164 (%)	n = 19 (%)	n = 36 (%)	n = 125 (%)	p-value*

**Age at diagnosis (y)**						**<0.001**
Median (Range)	53 (19–91)	46 (19–76)	55 (23–75)	67 (21–88)	68 (20–91)	
< 60	212 (62)	150 (91)	11 (58)	10 (28)	41 (33)	
≥ 60	132 (38)	14 (9)	8 (42)	26 (72)	84 (67)	

**Sex**						**0.003**
Male	270 (78)	141 (86)	13 (68)	22 (61)	94 (75)	
Female	74 (22)	23 (14)	6 (32)	14 (39)	31 (25)	

**Year of diagnosis**						**0.003**
2005–2009	29 (8)	23 (14)	0 (0)	2 (6)	4 (3)	
2010–2014	75 (22)	40 (24)	6 (32)	5 (14)	24 (19)	
2015–2019	158 (46)	75 (46)	7 (36)	15 (42)	61 (49)	
2020–2022	82 (24)	26 (16)	6 (32)	14 (38)	36 (29)	

**Race**						**<0.001**
White	230 (67)	90 (55)	16 (85)	28 (77)	96 (77)	
Black / African American	59 (17)	44 (27)	1 (5)	5 (14)	9 (7)	
Other	34 (10)	23 (14)	1 (5)	1 (3)	9 (7)	
Unknown	21 (6)	7 (4)	1 (5)	2 (6)	11 (9)	

**Ethnicity**						0.143
Hispanic/Latino	114 (33)	62 (38)	2 (11)	13 (36)	37 (30)	
Non-Hispanic/Latino	206 (60)	94 (57)	16 (84)	19 (53)	77 (62)	
Other/Unknown	24 (7)	8 (5)	1 (5)	4 (11)	11 (8)	

**ECOG Performance Status**						0.773
≤ 1	227 (66)	104 (64)	13 (68)	25 (69)	85 (68)	
2+	74 (22)	35 (21)	2 (11)	9 (25)	28 (22)	
Unknown	43 (12)	25 (15)	4 (21)	2 (6)	12 (10)	

**Ann Arbor Stage**						0.071
I	52 (15)	15 (9)	4 (21)	6 (16)	27 (22)	
II	33 (10)	18 (11)	0 (0)	2 (6)	13 (10)	
III	22 (6)	13 (8)	0 (0)	4 (11)	5 (4)	
IV	227 (66)	114 (70)	13 (68)	24 (67)	76 (61)	
Unknown	10 (3)	4 (2)	2 (11)	0 (0)	4 (3)	

**Nodal Disease**						**0.046**
Yes	215 (63)	112 (68)	8 (42)	23 (64)	72 (58)	
No	112 (33)	44 (27)	11 (58)	13 (36)	44 (35)	
Unknown	17 (4)	8 (5)	0 (0)	0 (0)	9 (7)	

**Extranodal Disease**						0.837
Yes	316 (92)	149 (91)	17 (89)	33 (92)	117 (94)	
No	24 (7)	12 (7)	2 (11)	3 (8)	7 (5)	
Unknown	4 (1)	3 (2)	0 (0)	0 (0)	1 (1)	

**LDH Elevation**						**0.009**
>1x ULN	177 (51)	100 (61)	7 (37)	21 (58)	49 (39)	
Not Elevated	124 (36)	48 (29)	7 (37)	13 (36)	56 (45)	
Unknown	43 (13)	16 (10)	5 (26)	2 (6)	20 (16)	

**IPI at diagnosis, mean (SD)**	2.51 (1.32)	2.50 (1.14)	2.38 (1.39)	2.88 (1.34)	2.42 (1.52)	0.423

***MYC* Rearrangement**						**0.045**
+	45 (13)	26 (16)	2 (11)	7 (19)	10 (8)	
−	33 (10)	9 (5)	2 (11)	6 (17)	16 (13)	
Unknown	266 (77)	129 (79)	15 (78)	23 (64)	99 (79)	

**CNS Involvement**						0.529
Yes	11 (3)	6 (4)	0 (0)	0 (0)	5 (4)	
No	329 (96)	155 (95)	19 (100)	36 (100)	119 (95)	
Unknown	4 (1)	3 (1)	0 (0)	0 (0)	1 (1)	

**Head/Neck Involvement**						**0.008**
Yes	94 (27)	53 (32)	2 (11)	3 (8)	36 (29)	
No	246 (72)	108 (66)	17 (89)	33 (92)	88 (70)	
Unknown	4 (1)	3 (2)	0 (0)	0 (0)	1 (1)	

**EBV+ by LMP or EBER**						**<0.001**
+	194 (56)	25 (15)	5 (26)	23 (64)	59 (47)	
−	112 (33)	118 (72)	13 (68)	13 (36)	50 (40)	
Unknown	38 (11)	21 (13)	1 (5)	0 (0)	16 (13)	

**Chemo Regimen in 1L** **						0.073
CHOP/CHOP-like	44 (14)	17 (10)	1 (5)	10 (28)	16 (13)	
EPOCH	220 (64)	110 (67)	15 (79)	19 (53)	76 (61)	
Hyper-CVAD or CODOX-M/IVAC	26 (8)	17 (10)	1 (5)	0 (0)	8 (6)	
Other	19 (6)	9 (5)	1 (5)	2 (6)	7 (6)	

**Additional Agents Used in 1L**						**0.028**
Proteasome Inhibitor	105 (31)	47 (29)	5 (26)	8 (22)	45 (36)	
Rituximab	60 (17)	31 (19)	4 (21)	11 (31)	14 (11)	
Daratumumab	12 (4)	7 (4)	2 (11)	2 (6)	1 (1)	

**CNS PPX in 1L**						**0.024**
IT MTX and/or AraC	90 (26)	50 (30)	2 (11)	5 (14)	33 (26)	
HD MTX	23 (7)	16 (10)	0 (0)	0 (0)	7 (6)	
None	179 (52)	74 (45)	11 (58)	25 (69)	69 (55)	
Unknown	52 (15)	24 (15)	6 (31)	6 (17)	16 (13)	

**Consolidative RT in 1L**						**0.014**
Yes	63 (18)	21 (13)	3 (16)	6 (17)	33 (26)	
No	270 (78)	142 (86)	14 (74)	30 (83)	84 (68)	
Unknown	11 (3)	1 (1)	2 (10)	0 (0)	8 (6)	

**Consolidative ASCT in 1L**						0.580
Yes	27 (8)	13 (8)	0 (0)	4 (11)	10 (8)	
No	307 (89)	150 (91)	17 (89)	32 (89)	108 (86)	
Unknown	10 (3)	1 (1)	2 (11)	0 (0)	7 (6)	

* Bolded p-values are statistically significant. P-values do not account for missing data rows. SD = Standard deviation.

** More detailed description in Supplemental Table 1.

**Table 2.** Survival Analysis using multiply imputed data

Risk Factor	Univariable Analysis, OS			Cox Regression (Multiple Imputation), OS		

	HR	95% CI	p-value*	HR	95% CI	p-value*

Cohort (vs HIV-PBL)						
PTLD-PBL	1.51	0.82–2.78	0.187	1.91	0.92–3.99	0.083
IC-PBL	1.17	0.83–1.64	0.370	0.80	0.47–1.37	0.418
OIS-PBL	1.59	0.99–2.54	0.055	0.93	0.49–1.79	0.832

Age (years)	1.02	1.01–1.03	**<0.001**	1.02	1.00–1.03	**0.018**

Female sex	1.52	1.08–2.15	**0.018**	1.26	0.84–1.88	0.263

Year of diagnosis (vs 2005–2009)						
2010–2014	1.49	0.79–2.81	0.220	1.10	0.50–2.4	0.817
2015–2019	1.88	1.02–3.46	**0.042**	1.65	0.79–3.41	0.180
2020–2022	1.61	0.83–3.12	0.162	1.51	0.67–3.38	0.322

Race (vs White)						
Black/African American	1.58	1.09–2.30	**0.016**	1.43	0.91–2.23	0.118
Other	0.49	0.24–1.00	**0.049**	0.63	0.28–1.40	0.253

Ethnicity (vs Hispanic/Latino)						
Non-Hispanic/Latino	1.35	0.96–1.89	0.087	1.08	0.72–1.62	0.717

ECOG 2+ (vs <2)	2.59	1.82–3.68	**<0.001**	1.91	1.14–3.2	**0.014**

Stage III/IV (vs I/II)	2.52	1.64–3.86	**<0.001**	1.75	1.02–3.02	**0.043**

Extranodal disease	1.57	1.16–2.13	**0.004**	1.27	0.84–1.93	0.258

LDH elevation	3.04	2.12–4.36	**<0.001**	2.63	1.65–4.20	**<0.001**

IPI (per one point increase)	1.45	1.25–1.67	**<0.001**	1.05	0.84–1.32	0.643

*MYC* rearrangement	1.34	0.27–6.68	0.712	1.26	0.26–6.06	0.761

EBV+ by either LMP1 or EBER	0.59	0.42–0.84	**0.004**	0.71	0.46–1.10	0.126

1L Regimen (vs CHOP)						
EPOCH	0.77	0.49–1.21	0.254	0.65	0.39–1.07	0.091
High-Intensity	1.01	0.52–1.97	0.983	1.36	0.62–3.01	0.444
Other	1.33	0.64–2.76	0.448	0.89	0.43–1.83	0.742

PI in 1L	1.05	0.76–1.46	0.766	0.99	0.68–1.43	0.950

CNS PPx (vs none)						
IT MTX/AraC	0.76	0.52–1.10	0.149	0.81	0.51–1.28	0.363
HD IV MTX	0.87	0.50–1.52	0.621	0.69	0.34–1.39	0.295

Consolidative RT in 1L	0.60	0.38–0.93	**0.024**	0.93	0.57–1.52	0.768

* Bolded p-values are statistically significant

## Data Availability

De-identified patient data is available upon request from the corresponding author and/or senior author, via email.
